# COVID-19 as a Possible Cause of Functional Exhaustion of CD4 and CD8 T-cells and Persistent Cause of Methicillin-Sensitive Staphylococcus aureus Bacteremia

**DOI:** 10.7759/cureus.9000

**Published:** 2020-07-04

**Authors:** Ivana Choudhury, Haowei Han, Kaushik Manthani, Sandeep Gandhi, Rameshchandra Dabhi

**Affiliations:** 1 Family Medicine, Kaweah Delta Health Care District, Visalia, USA; 2 Family Medicine, Peconic Bay Medical Center-Northwell Health, Riverhead, USA; 3 Medicine, Peconic Bay Medical Center-Northwell Health, Riverhead, USA

**Keywords:** covid 19, mssa bacteremia, infectious endocarditis, aortic root abscess

## Abstract

We report a case of a 73-year-old male with a history of diabetes mellitus, osteomyelitis, methicillin-sensitive Staphylococcus aureus (MSSA) bacteremia who recently completed an extended intravenous course of cefazolin eight days back, and presented with MSSA bacteremia complicated by epidural abscess, endocarditis, and aortic root abscess. Meanwhile, the patient was tested positive for severe acute respiratory syndrome coronavirus 2 (SARS-CoV-2) by reverse transcription-polymerase chain reaction (RT-PCR). Even with aggressive antibiotic treatment, the patient remained bacteremic and developed endocarditis with a worsening aortic root abscess. We suspect coronavirus disease 2019 (COVID-19) as a cause for the infectious paradox and will discuss the possible mechanisms in this case report.

## Introduction

The current coronavirus disease 2019 (COVID-19) pandemic has burdened the modern health care system. Similar to severe acute respiratory syndrome coronavirus (SARS-CoV) and the Middle East respiratory syndrome (MERS), SARS-CoV-2 is primarily known to cause ailments to the human respiratory system. Other complications have been reported, including cardiovascular disease, renal failure, and sequelae of a hypercoagulable state [1-3]. To our current knowledge, there is sparse clinical evidence suggesting the correlation between COVID-19 infection and its effect on the human immune system, making patients more susceptible to certain infections.

Although cytokines and immune regulation are vital for body response to infection, dysregulation or excessive responses may cause harm. There is a positive correlation between cytokine storm and disease severity. High expression of Th1 and Th2 cytokines has been detected in COVID-19 patients which accounts for acute respiratory distress syndrome (ARDS), multi-systemic organ failure, and high rate of mortality and morbidity [4]. However, the correlation between COVID-19 immune exhaustion and mortalities from infectious etiologies have not been extensively reported to date.

## Case presentation

A 73-year-old male recently treated for methicillin-sensitive Staphylococcus aureus (MSSA) bacteremia secondary to presumed source of non-healing diabetic foot ulcer (completed a six weeks course of intravenous (IV) cefazolin eight days ago, negative transesophageal echocardiogram (TEE)) presented to our emergency department with lower back pain with extension to the left flank region, urinary incontinence, and an altered mental status. Other past medical and past surgical history was remarkable for diabetes mellitus, chronic foot osteomyelitis, aortic stenosis (status post transcatheter aortic valve replacement (TAVR) in 2003 later replaced with a bioprosthetic aortic valve (BAVR) in 2017), and persistent atrial fibrillation (on warfarin). Initial vital signs: temperature: 100.2 °F, blood pressure: 113/71 mmHg, pulse: 99 beats/min, respiratory rate: 24/min, oxygen saturation: 99% on room air. Physical exam was significant for two right lower extremity foot ulcers that did not have any surrounding erythema, necrosis or other signs of infection. No splinter hemorrhage, Osler’s nodes, or Janeway lesions were noted. 

Laboratory results were as follows: white blood cell (WBC) count: 13.6 (10^3/µL) with neutrophilia, glucose: 220 mg/dL, magnesium: 1.5 mg/dL, lactate: 2.7 mg/dL, aspartate transaminase: 99 units/L, erythrocyte sedimentation rate: 30 mm/hr, C-reactive protein: 28.86 mg/L, procalcitonin: 8.25 ng/mL. Urinalysis was remarkable for large blood, protein, negative WBC esterase, nitrite, and but had few bacteria. Computer tomography (CT) of the abdomen/pelvis revealed possible recent passing of a stone, cystitis, and pyelonephritis. The patient was admitted to the hospital for additional workup.

Due to concern of multi-drug resistant infections, IV vancomycin and piperacillin/tazobactam were initiated, as well as IV fluids per sepsis protocol. Meanwhile, the patient was also found to be positive for SARS-CoV-2 by reverse transcription-polymerase chain reaction (RT-PCR) during admission and was started on a course of hydroxychloroquine. His blood cultures grew MSSA, and the patient was restarted on IV cefazolin subsequently. 

**Figure 1:**
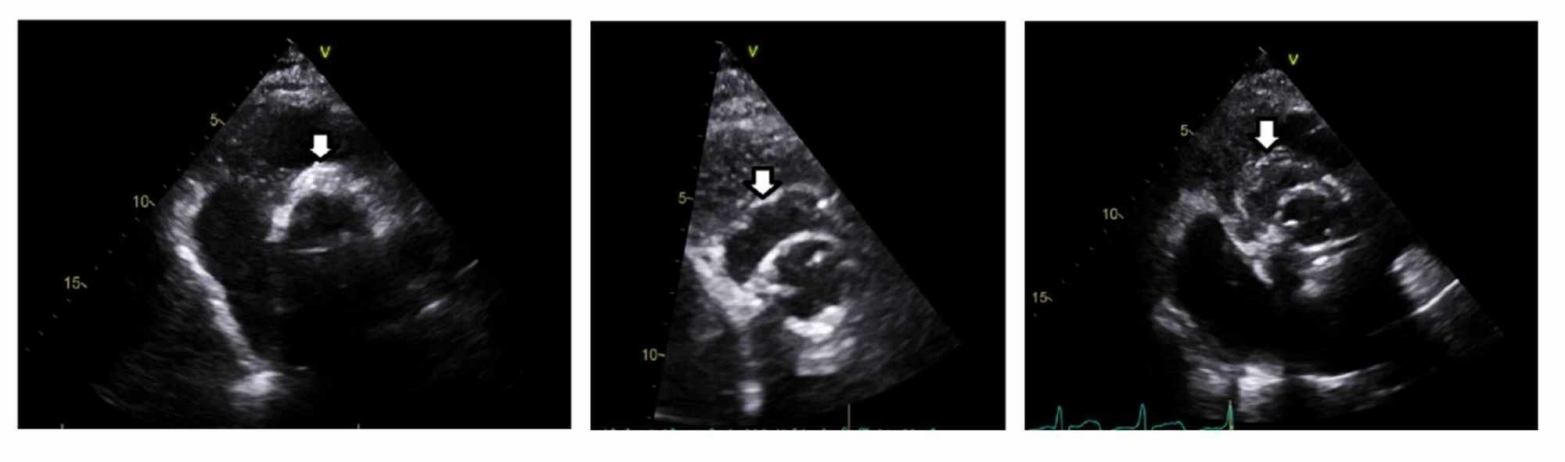
Transthoracic echocardiogram, parasternal, short axis view, aortic valve Leftmost image: increased echogenicity of the annulus of the bioprosthetic aortic valve, but no definite abscess (arrow). Middle image: development of paravalvular abscess. Rightmost image: echolucent area, suggestive of an increase in the abscess size.

**Figure 2:**
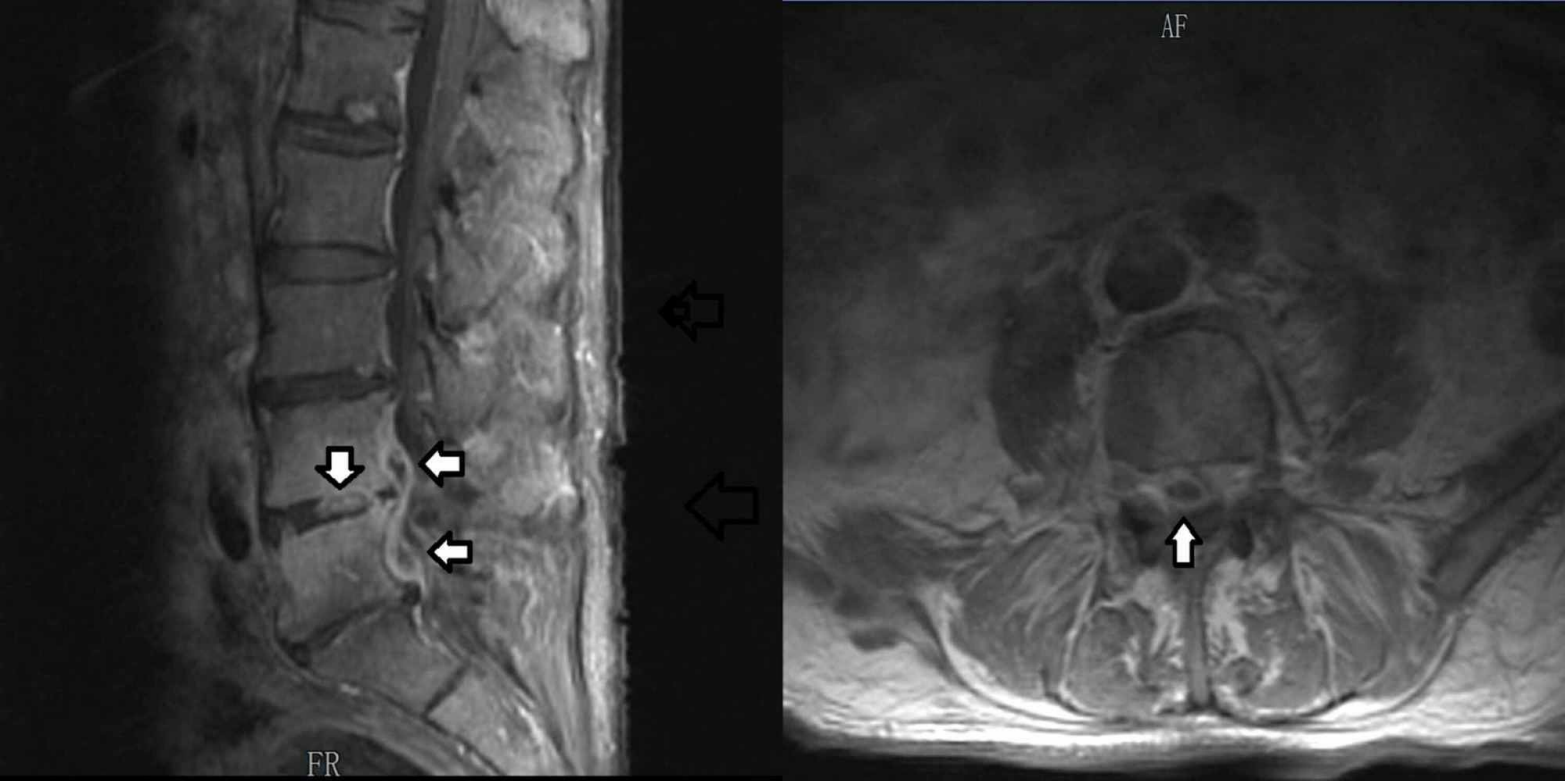
MRI of the L-spine Coronal view (left) and transverse view (right) of the spinal epidural abscess.

**Figure 3:**
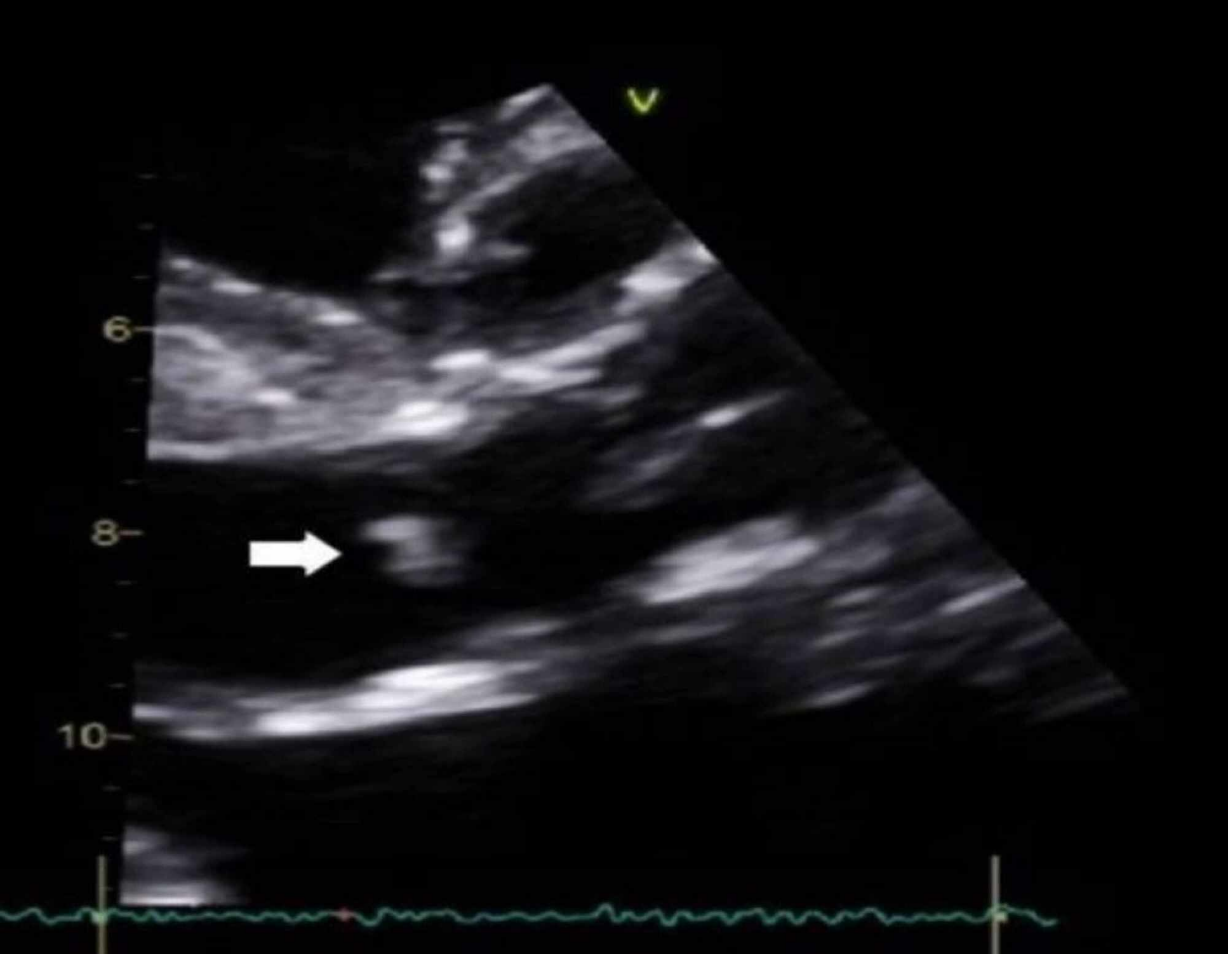
Aortic valve endocarditis as seen on the transthoracic echocardiogram

The patient’s initial transthoracic echocardiogram (TTE) was remarkable for a bioprosthetic aortic valve with normal mobility (Figure 1). Per the modified Duke’s criteria for diagnosing infective endocarditis (IE), the patient met one major criteria (positive blood cultures), which gives him a diagnosis of definite IE. Upon reviewing the patient’s TEE two weeks prior at the other facility, there was no evidence of cardiac valve vegetation, and the patient had a normal ejection fraction. Daily blood cultures continued to grow MSSA and hence, the antibiotic was changed to IV daptomycin. Magnetic resonance imaging (MRI) of the lumbar spine (L-spine) revealed an epidural abscess and concurrent osteomyelitis at the vertebral levels of L4-L5 (Figure 2). Surgical intervention was not pursued due to the patient's positive COVID infection and underlying comorbidities. A repeat TTE performed several days later revealed vegetation on a bioprosthetic aortic valve (as seen in Figure 3) with periannular abscess extending up to the root and ascending aorta (Figure 1). According to modified Duke’s criteria, the patient now met two major criteria for definitive IE (echocardiographic evidence of vegetation on the bioprosthetic aortic valve, and two positive blood cultures indicative of IE). Oral rifampin was added to the IV daptomycin. The day when oral rifampin was added, the patient’s blood cultures showed no growth of bacteria. Therefore it is unknown if rifampin played a role in the patient’s clearance of MSSA from the blood. 

During his hospital stay, the patient then retested positive for COVID-19, and was started on oral ciprofloxacin and IV ceftaroline. Several days later, the patient tested negative for COVID-19. 

A repeat MRI L-spine revealed marginal improvement of the epidural abscess. The patient was discharged to subacute rehabilitation with oral rifampin and IV daptomycin. Unfortunately, two weeks later the patient was readmitted for chest pain, and TTE revealed an increase in the size of the aortic root abscess with new-onset systolic dysfunction (ejection fraction of 30%) (Figure 1). Due to the extensive risks associated with surgical management of the abscess and replacement of the valve, the patient and his family determined that hospice care was most appropriate.

## Discussion

Spinal epidural abscess (SEA) is an uncommon pyogenic infection of the central nervous system primarily affecting patients above 50 years and is primarily treated with surgical decompression and abscess drainage along with prolonged antibiotic therapy [5,6]. Risk factors include alcohol abuse, obesity, chronic kidney disease, diabetes mellitus, injection drug use, or indwelling catheter use [6]. Clinical manifestations of SEA include back pain (nontraumatic or traumatic), neck pain, paresthesia, radiculopathy, focal neurological deficits, bowel or bladder incontinence, fevers, acute mental status changes [6]. In a retrospective, case-control study measuring patients diagnosed with SEA, all portions of the vertebral spine may be affected, with the majority of cases occurring in the lumbar spine. In this study, 39.5% of all cases only received antibiotic therapy, while 61% underwent neurological drainage and debridement of the SEA [6]. Empirical antibiotics should cover MSSA, methicillin-resistant Staphylococcus aureus (MRSA), and Streptococci [6]. 

Meanwhile, despite receiving IV antibiotics, our patient progressed into aortic valve abscess and endocarditis. In one prospective cohort study involving 556 patients, prosthetic valve endocarditis (PVE) is present 20% of the time in patients with IE. The most common causative organism is Staphylococcus aureus (23% of patients), followed by coagulase-negative Staphylococcus (16.9% of patients) [7]. Health care-associated PVE occurred in 36.5% of patients in this cohort study, while 71% of health care-associated PVE occurred within the first year of TAVR placement [7]. 

It was also found that persistent bacteremia or a hospital-acquired nosocomial infection is strongly linked with Staphylococcus aureus IE, with nosocomial infection defined as developing IE after 48 hours of hospitalization with no previous signs or symptoms of IE [7]. Despite having a negative TTE and TEE prior to visualizing the PVE, it is important to note that persistent MSSA bacteremia was likely the strongest risk factor in his development of a PVE. However, it is important to discuss if the patient’s positive COVID-19 infection played a role in his immune system’s ability to fight off the MSSA infection. 

In one prospective study of 72 patients, patients with IE associated with MSSA infection significantly had a higher rate of major embolism than MRSA patients, as well as a significantly more unknown origin of bacteremia [8]. IE complicated MRSA infection is usually associated with nosocomial origin, surgical site infection, prior surgery within six months, or persistent bacteremia in the setting of a catheter [8]. However, the mortality rate was higher in patients with MRSA than in patients with MSSA.

To protect itself from the host’s immune system, bacteria can protect itself with biofilm formation on an implanted or indwelling device, particularly Staphylococcus aureus and Staphylococcus epidermis [9,10]. While it does not have proven efficacy against biofilm-encoated bacteria, rifampin may adequately penetrate biofilms while reducing the adherence of bacteria to surfaces [10]. While rifampin is not recommended under American Heart Association guidelines for the treatment of IE, one retrospective cohort study involving 84 patients found that many clinicians are more likely to add rifampin to the standard antibiotic therapy for the treatment of MSSA IE in patients who have prolonged bacteremia or appear severely ill [11]. 

Multiple recent clinical trials in vitro suggest that COVID-19 causes a functional exhaustion of cluster of differentiation (CD)8 T-cells and natural killer (NK) cells due to persistent stimulation from the virus, thus inducing T-cell exhaustion [12-14]. A retrospective study in Wuhan, China found that 19 patients in the intensive care unit (ICU) confirmed to have COVID-19 had markedly reduced CD4 and CD8 T-cells [12]. Cytotoxic T lymphocytes (CTL) are part of the immune system response that play a role against viral pathogens. CD8 T-cells secrete interferons (IFN-γ), perforin and granzymes to eradicate viruses, while CD4+ helper T cells enhance CD8 cells and B cells to help them clear the viral pathogen [12,13]. CD4 cells are vital to the role of CD8 T-cells and B-cells for cellular and humoral immune responses, so in patients with cancer or chronic infections, both CD4 and CD8 T-cells can become exhausted and lose function [15]. For a patient with MSSA bacteremia and COVID-19, such as ours, CD4 and CD8 T-cell functional exhaustion may be why our patient required an extended course of IV antibiotic therapy. Unfortunately, we didn’t check this patient’s CD4/CD8 counts during his hospital course. While further research is needed, it is important to monitor a COVID-19 positive patient being treated for bacteremia, spinal epidural abscess, or cardiac valvular vegetations, as COVID-19 may cause an immunocompromised state, thus making the patient more susceptible to bacterial infections. 

Overall, this patient presented with recurrent bacteremia and multiple organ infections. Although the diabetic ulcers may have served as the source of the MSSA infection, due to the noninfectious site of the wound, we suspect that the cause is a reactivated bacteremia due to a dual immunocompromised state secondary to diabetes mellitus and COVID-19. We suspect this because it is unusual to have a PVE after 12 months of a bioprosthetic aortic valve replacement, and it is also unusual to have multiple abscesses occur eight days after stopping IV antibiotics.

## Conclusions

Our patient presented with a recurrent and persistent MSSA bacteremia and osteomyelitis, complicated by a spinal epidural abscess, bioprosthetic valve endocarditis and aortic root abscess despite appropriate antibiotic therapy. In COVID-19 patients, we see many patients’ hospital courses complicated by cytokine storms and end-stage organ failure. However, clinicians should remember that COVID-19 causes an immunocompromised state due to the functional exhaustion of CD4 and CD8 T-cells. Our case has suggested further research and observational studies into the correlation between COVID-19 and other infectious processes.

## References

[1] Bansal M (2020). Cardiovascular disease and COVID-19. Diabetes Metab Syndr.

[2] Durvasula R, Wellington T, Mcnamara E, Watnick S (2020). COVID-19 and kidney failure in the acute care setting: our experience from seattle. Am J Kidney Dis.

[3] Becker RC (2020). COVID-19 update: Covid-19-associated coagulopathy. J Thromb Thrombolysis.

[4] Ye Q, Wang B, Mao J (2020). The pathogenesis and treatment of the 'Cytokine Storm' in COVID-19. J Infect.

[5] Saito K, Fukazawa R, Ogura S, Kasai T, Mizuno T (2019). A case of extensive epidural abscess concomitant with intracranial involvement due to Staphylococcus aureus successfully treated with ceftriaxone in combination with linezolid and rifampin. ENeurologicalSci.

[6] Artenstein AW, Friderici J, Holers A, Lewis D, Fitzgerald J, Visintainer P (2016). Spinal Epidural Abscess in Adults: A 10-Year Clinical Experience at a Tertiary Care Academic Medical Center.

[7] Wang A, Athan E, Pappas PA (2007). Contemporary clinical profile and outcome of prosthetic valve endocarditis. JAMA.

[8] Hill EE, Peetermans WE, Vanderschueren S, Claus P, Herregods MC, Herijgers P (2008). Methicillin-resistant versus methicillin-sensitive Staphylococcus aureus infective endocarditis. Eur J Clin Microbiol Infect Dis.

[9] Zheng Z, Stewart PS (2002). Penetration of rifampin through Staphylococcus epidermidis biofilms. Antimicrob Agents Chemother.

[10] Croes S, Beisser PS, Neef C, Bruggeman CA, Stobberingh EE (2010). Unpredictable effects of rifampin as an adjunctive agent in elimination of rifampin-susceptible and -resistant Staphylococcus aureus strains grown in biofilms. Antimicrob Agents Chemother.

[11] Riedel DJ, Weekes E, Forrest GN (2008). Addition of rifampin to standard therapy for treatment of native valve infective endocarditis caused by Staphylococcus aureus. Antimicrob Agents Chemother.

[12] Diao B, Wang C, Tan Y (2020). Reduction and functional exhaustion of t cells in patients with coronavirus disease 2019 (COVID-19). Front Immunol.

[13] Ganji A, Farahani I, Khansarinejad B, Ghazavi A, Mosayebi G (2020). Increased expression of CD8 marker on T-cells in COVID-19 patients. Blood Cells Mol Dis.

[14] Zheng M, Gao Y, Wang G (2020). Functional exhaustion of antiviral lymphocytes in COVID-19 patients. Cell Mol Immunol.

[15] Kamphorst AO, Ahmed R (2013). CD4 T-cell immunotherapy for chronic viral infections and cancer. Immunotherapy.

